# Association of HLA-B27 and Behcet’s disease: a systematic review and meta-analysis

**DOI:** 10.1186/s13317-019-0112-x

**Published:** 2019-03-19

**Authors:** Alireza Khabbazi, Leila Vahedi, Morteza Ghojazadeh, Fariba Pashazadeh, Amin Khameneh

**Affiliations:** 1 Connective Tissue Diseases Research Center, Tabriz University of Medical Sciences, Tabriz, Iran; 2 Liver and Gastrointestinal Disease Research Centre, Tabriz University of Medical Sciences, Tabriz, Iran; 3 Medical Faculty, Research Center for Evidence Based Medicine, Tabriz University of Medical Sciences, Tabriz, Iran; 4 Research Center for Evidence Based Medicine, Tabriz University of Medical Sciences, Tabriz, Iran; 5 Tabriz Faculty of Medicine, Tabriz University of Medical Sciences, Tabriz, Iran

**Keywords:** Behcet’s disease, HLA-B27, Correlation, Case control study

## Abstract

**Background:**

To calculate the genetic impact of the “HLA-B27” allele on the risk of Behcet’s disease (BD) progression using a systematic review and meta-analysis on case control papers.

**Methods:**

A systematic review search was conducted on the MeSH keywords of Behcet’s disease, HLAB27 and B27 in PubMed, Scopus, ProQuest, EMBASE, SID, Magiran, IranDoc and IranMedex databases from 1975 to Aug 2017. Data underwent meta-analysis (random effect model) in CMA2 software. Pooled odds ratios with 95% confidence intervals were calculated for each study. The heterogeneity of the articles was measured using the I^2^ index.

**Results:**

Twenty two articles met the inclusion criteria for 3939 cases and 6077 controls. The pooled OR of “HLA-B27” in BD patients compared with controls was [1.55 (CI 95% 1.01–2.38), P = 0.04]. The OR differ among different countries or geographical areas, focus on domination the European countries. Quality of studies was moderate and heterogeneity was relatively high (I^2^ = 66.9%).

**Conclusions:**

There is a significant correlation between HLA-B27 and Behcet’s Disease, but it was weak. Environmental and genetic factors might determine which the “HLA-B27” alleles manifest Behcet’s disease progression. Future researches is required to perform about what factors can do to positively and separately influence Behcet’s disease.

## Introduction

Behcet’s disease (BD) is a recurrent inflammatory disease characterized by four main symptoms, including recurrent oral ulcers, genital ulcers, skin lesions and uveitis [1, 2]. BD has been spread worldwide; however, it is observed more commonly along the Silk Road [3]. The etiology and pathogenesis of BD is unknown [2, 4]. However, environmental and genetic factors are important agents in the development of the disease [2, 3]. Complex HLA/MHC is a genetic region with a biologically important action that is strongly associated with autoimmune diseases such as BD, ankylosing spondylitis (AS) and reactive arthritis [3, 4]. Although MHC class I specially “HLA-B5/B51” have the strongest associations with BD; data about “HLA-B27”are conflicting and may increase susceptibility to BD [2, 5, 6]. HLA-B27” is one of the attractive issues in medicine that play an important role in the pathogenesis of diseases like seronegative spondyloarthropathies and have a protective role in some infections [2, 7]. The frequency of “HLA-B27” varies among populations which may be the result of genetic and environmental factors [8, 9]. Furthermore, linkages between HLA-B alleles with other MHC and non-MHC genes could alter the penetration and clinical expression of the disease [7, 10]. Although these antigens are not as diagnostic criteria but are used for the conforming of diagnosis and the assessment of complications [10]. We conducted a systemic review and meta-analysis on case–control articles in order to the evolution of Behcet disease–gene association with the goal calculating the risk increase for BD progression related to “HLA-B27” and comparing among across the continents.

## Methods and materials

Case–control articles in which the “HLA-B27”related to BD were searched by electronic searches of the PubMed, Scopus, ProQuest, EMBASE, SID (Scientific Information Data 5 IranDoc (Iranian Research Institute for Information Science and Technology), Magiran and IranMedex electronic databases from January 1975 to Aug 2017 using key words of Behcet’s disease, Behcet’s syndrome, HLA-B27, B27, B*27 and their combinations. The Preferred Reporting Items for Systematic Reviews (PRISMA) checklist and PICO approach (participants: population with Behcet’s disease; interventions: genetic background for positive HLA-B27; comparison: patients with BD and control groups) [11] used for the search. References of the related review studies were assessed manually. In addition, unpublished studies and documents (grey literatures), and studies offered at congresses were scanned. In the item of unpublished studies or ambiguous data, we contacted the authors to gain further information. Besides, because of the rareness of BD in certain areas, a number of articles were also included that did not conform to the PRISMA checklist to allow coverage of specific items in the results. Papers designed as a case–control study and had acceptable information to create a 2*2 table in BD patients and controls for the frequency of “HLA-B27” were included the study. Moreover, all subtypes of “HLA-B27” with or without concomitance with other kinds of HLA class I alleles and all ages, genders, countries and ethnicities were reflected as inclusion criteria. Studies recording animal samples, case reports, case series, letters, disagreements between the authors, as well as studies in which a subset of BD patients (such as ankylosing spondylitis and uveitis) were excluded. This study has been conducted at Tissue Disease Research Center (TDRC) of the Tabriz University of Medical Sciences/Iran.

### Eligible studies and data extraction

All articles were independently assessed by two appraisals (K.A. and V.L.). Any disagreement between the authors was referred to third author (G.M.) for eligibility. Extraction from each 6 article was conducted independently by two authors. The following data were collected: the name of first author, published date, study population location, BD samples, control samples and frequency of HLA-B27-positive cases and controls. Then countries divide to four groups, including of the Far East, Middle East, Europe and Africa. Nonetheless Turkey is geographically portion of Europe; however, it was included in the Middle East because of genetic matters of population.

### Statistical analysis

Odds ratio (OR) with 95% confidence interval (95% CI) were measured for all studies. Data were based on pooled to compare the frequency of “HLA-B27” between BD cases and controls using a random effect model of meta-analysis.

Heterogeneity between studies was measured using Cochran’s Q and I^2^ tests to determinate the percentage of changes among studies using the software CMA v.2.0.

To survey of publication bias Funnel plot analysis was used for investigating the publication bias. Moreover, publication bias was evaluated using the Egger’s test. The results of the metaanalysis were presented as forest plots. All statistical tests were 2-tailed and a p-value lesser 0.05 was considered statistically significant. Endnote X5 was used to classify the data, review the titles and abstracts, as well as identify duplicated studies.

### Ethics statement

The protocol of the study was approved by the Ethics Committee of Tabriz University of Medical Sciences and all data were kept without stating the patients’ names and addresses.

## Results

**Fig. 1:**
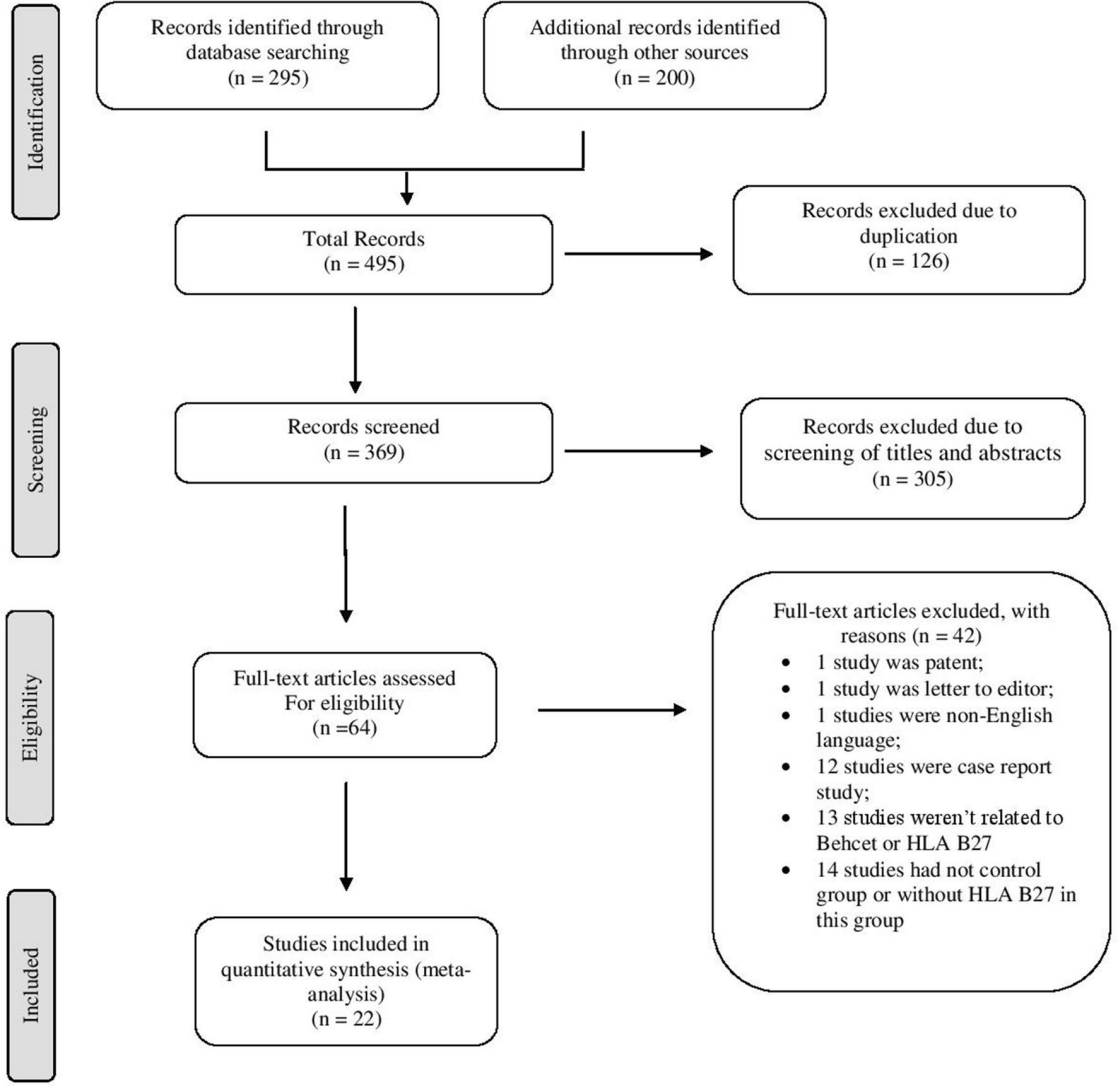
Flow diagram of studies for inclusion in the systematic review and meta-analysis

The flow chart of systematic review is shown in Fig. 1. A total of 495 papers, 126 papers were 7 excluded as duplication, 305 papers were excluded after reviewing titles and abstracts, and 42 papers dies after reviewing full texts. A final total of 22 articles met the inclusion criteria for the meta-analysis that the characteristics of papers [2, 4, 5, 10, 12–29] are shown in Table 1.

**Table 1:** Characteristics of studies (case and control groups) with Behcet disease through the world and continents

Authors	Places	Cases N = 22 papers		Controls N = 22 papers		Effect size and 95% CI	Heterogeneity		
		Sample size (F %)	HLA-B27 (F)	Sample size (F %)	HLA-B27 (F)	OR % (lower limit–upper limit), P-value	df (Q)	P-value	I^2^
Far East									
Takano et al. [12]	Japan	73 (43.5)	0	141 (45.6)	3	–	–	–	–
Chung et al. [13]	Republic of China	51 (30.4)	4	128 (41.4)	6	–	–	–	–
Cibo et al. [14]	China	44 (26.2)	9	40 (12.9)	12	–	–	–	–
Subtotal		168 (4.3)	13	309 (5.1)	21	0.82% (0.36–1.86), *P *= 0.6	2	0.33	7.63
Middle East									
Brautbar et al. [15]	Israel	24 (0.8)	0	615 (16.9)	28	–	–	–	–
Yazici et al. [16]	Turkey	36 (1.3)	5	86 (2.4)	4	–	–	–	–
Muftuuglu et al. [17]	Turkey	119 (4.2)	7	268 (7.4)	9	–	–	–	–
Gul et al. [2]	Turkey	174 (6.1)	6	191 (5.3)	7	–	–	–	–
Pirim et al. [18]	Turkey	75 (2.6)	7	54 (1.5)	6	–	–	–	–
Mizuki et al. [19]	Turkey	33 (1.2)	1	65 (1.8)	6	–	–	–	–
Ombrello et al. [20]	Turkey	1190 (41.7)	44	1257 (34.6)	42	–	–	–	–
Xavier et al. [21]	Iran	973 (34.1)	25	824 (22.7)	21	–	–	–	–
Al-Okaily et al. [22]	Saudi Arabia	60 (2.1)	1	60 (1.7)	1	–	–	–	
Mizuki et al. [23]	Turkey	172 (6.0)	43	212 (5.8)	12	–	–	–	
Subtotal		2856 (72.5)	139	3632 (59.8)	136	1.38% (0.83–2.32), *P *= 0.2	9	0.00	63.08
Europe									
Chamberlain [24]	UK	28 (5.1)	7	613 (34.4)	15	–	–	–	–
Lehnar et al. [25]	UK	65 (11.9)	8	300 (16.8)	17	–	–	–	–
Munoz-Saa et al. [26]	Spain	24 (4.4)	0	165 (9.2)	7	–	–	–	–
Subtotal		544 (13.8)	34	1784 (29.4)	57	2.86 (0.90–9.02), *P *= 0.07	5	0.00	77.46
Africa									
Bettencourt et al. [10]	Portugal	78 (14.3)	8	208 (11.7)	0	–	–	–	–
Piga et al. [4]	Italy	45 (8.3)	3	185 (10.4)	8	–	–	–	–
Montes-Cano et al. [27]	Spain	304 (55.9)	8	313 (17.5)	10	–	–	–	–
Subtotal		544 (13.8)	34	1784 (29.4)	57	2.86 (0.90–9.02), *P *= 0.07	5	0.00	77.46
Choukri et al. [28]	Morocco	86 (23.2)	3	116 (33.0)	8	–	–	–	–
Salky et al. [29]	Tunisia	165 (44.5)	0	124 (35.2)	4	–	–	–	–
Radouane et al. [5]	Morocco	120 (32.3)	16	112 (31.8)	3	–	–	–	–
Subtotal		371 (9.4)	19	352 (5.8)	15	1.44% (0.27–7.73), *P *= 0.6	2	0.03	71.15
Total		3939 (100)	205	6077 (100)	229	1. (1.01–2.38), *P *= 0.04	21	0.00	66.97

*OR* odds ratio, *CI* confidence interval, *df* degrees of freedom, *I*^*2*^ I-squared, *P* P-value, *F* Frequency, *%* Percentage

**Fig. 2:**
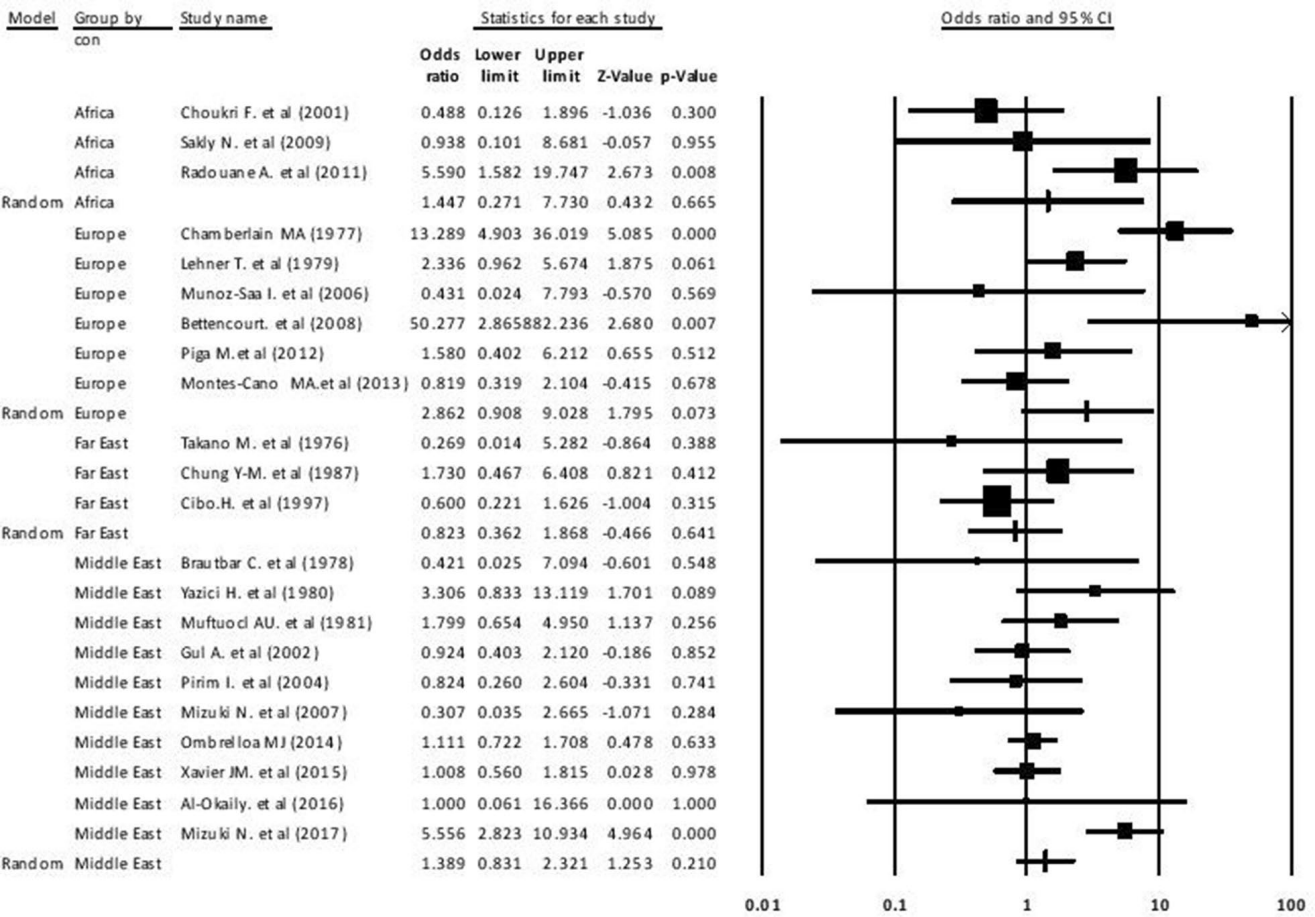
Forest plot of HLA-B27 in Behcet disease between case and control groups with a 95% confidence interval

The final data contained a total of 3939 cases and 6077 controls with a larger sample size for Xavier et al. from Iran [21]. Furthermore, a higher case and control populations related to the Middle East and a higher ratio control than case population related to the Europe. The pooled OR for BD susceptibility was 1.55 (95% CI 1.01–2.38) with significant difference (P = 0.04) that the results are shown in Fig. 2. In most areas, the pooled ORs for HLA-B27-positive to progress BD were > 1 through the countries. Comparison among continents illustrated that Europe had higher the pooled OR of “HLA-B27” than Africa, Middle East and Far East. The pooled OR was more than one for all continents exception the Far East (Table 2).

**Table 2:** Comparison the indicators of studies across continents

Group	Number studies	Effect size and 95% confidence interval				Test of null (2-tail)		Heterogeneity		
		OR	Lower limit	Upper limit	Z-value	P-value	Q-value	df (Q)	P-value	I-squared
Far East	3	0.82	0.36	1.86	0.46	0.64	2.16	2	0.33	7.63
Middle East	9	1.38	0.83	2.32	1.25	0.21	24.37	9	0.00	63.08
Europe	6	2.86	0.90	9.02	1.79	0.07	22.18	5	0.00	77.46
Africa	3	1.44	0.27	7.73	0.43	0.66	6.93	2	0.03	71.15
Total	22	1.55	1.01	2.38	2.01	0.04	63.58	21	0.00	66.97

**Fig. 3:**
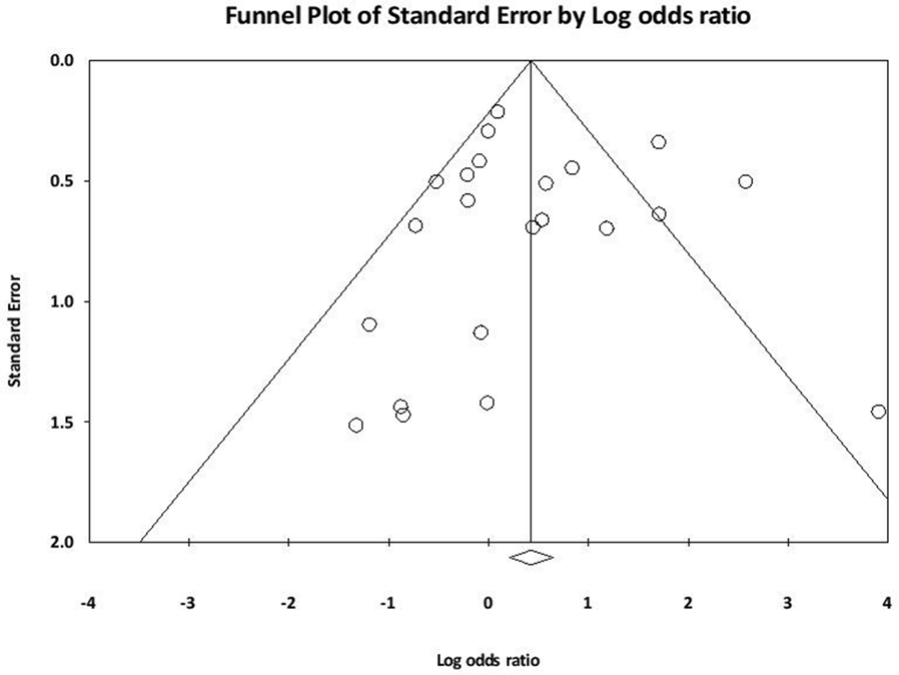
Distribution publication bias or funnel plot

**Fig. 4:**
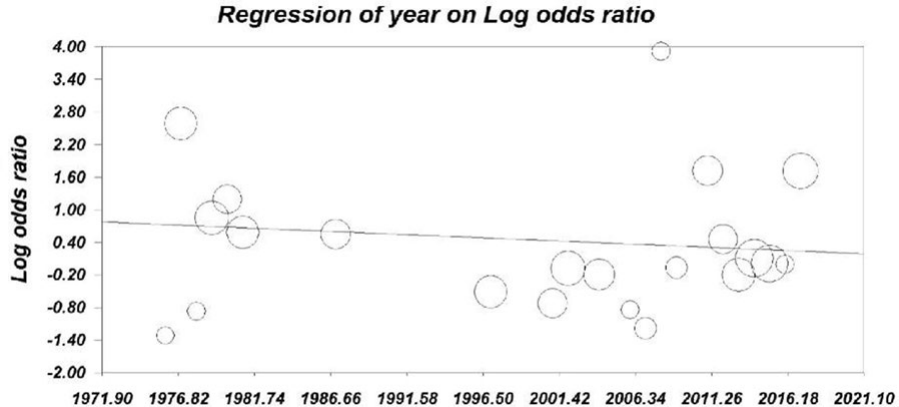
Meta Regression based on year of publication

The between-study heterogeneity was moderate (Total I^2^ = 66.97%), so the Random-Effect Model was the selected method for statistical analysis at this study. The tests with P50% stated significant heterogeneity. A funnel plot was used to detect publication bias in meta-analyses had a slightly asymmetric shape and Egger’s test (t-value = 0.006, df = 20, P-value = 0.99)) was not statistically significant (Fig. 3). In addition, Meta-regression was performed based on year of publication. The results of Metaregression showed that the slope of the regression line was not significant. Such that with an increase of 1 year, 0.012 units decreases the log odds ratio for the incidence of the disease (β = − 0.012, sd = 0.015, P-value = 0.44) (Fig. 4).

## Discussion

The pooled estimates of this meta-analysis of 3939 cases and 6077 controls indicate that the risk of “HLA-B27” for BD progression is increased by a factor of 1.55. In current study, 22 articles were included in a meta-analysis in order to assess the association between “HLA-B27” frequency and BD. To the best of our knowledge, this study is the first meta-analysis study which compare the frequency of “HLA-B27” in BD patient and healthy individuals. Previous studies have been performed on “HLA-B51/B5” which has been known as the strongest association in BD [22, 30] with an odds ratio of 5.78 in carriers than non-carriers [2]. However, recent studies showed new susceptibility genes in the rest regions of the HLA class I and numerous non-HLA genes for BD [20, 31, 32]. This study has been conducted regarding to the role other genes and it seems that there is a correlation between BD and “HLA-B27”; however, this correlation is weak in comparison with “HLA-B51/B5”.

Behcet’s disease is a type auto inflammatory disorder and numerous genetic and environmental factors rising interest to develop BD [3, 33]. Mc Gonagle et al. revealed that BD, psoriasis, psoriatic arthritis (PsA) and spondyloarthropathies (SpAs) can be considered to have a strongly shared immunopathogenetic basis [34]. All these diseases clinically overlapping together and associated with MHC class I (MHC-I) alleles such as “HLA-B51”, “HLA-B5”, “HLA-B27”and HLA-C0602, although these connections are stronger in some diseases [20, 21]. However, other class 1 alleles are also expected to be involved. Indeed, the risk of the HLA-B27 allele appears to be shared in all SpA and Behcet’s disease groups [20, 31].

In another study, Gül has stated that, despite the strong relationship of HLA-B51 with BD, there is a controversy about this relationship because of role of the other variants of MHC class I [35, 36]. In another comprehensive review has been emphasized on the role of other class 1 alleles in BD based on the results of recent studies [35]. Indeed, it seems that the role of “HLA-B51” on BD is unclear whether “HLA-B51” gene is BD susceptibility itself or other genes are effective in the disease [35, 36]. Therefore, this meta-analysis was conducted to gain information concerning relationship between BD and the HLA-B27 allele. As BD is a chronic and relapsing vasculitis, with involvement several body systems and significant morbidity and mortality [37] a comprehensive disease management is necessary to prevent or minimize the effects of the disease [3, 32]. On the other hand, due to progression in knowledge the pathogenesis of BD, and introduction immunologic agents as risk factors [3], a good management could perform according to pathogenesis of BD [3]. For instance, in patients with BD due to the lack of diagnostic pathognomonic laboratory tests, disease identified according to clinical criteria [32]. It is expected that these factors could be used as diagnostic criteria in the future.

As a consequence, on time diagnosis, appropriate treatments and good fallow up could reduce morbidity and mortality or improve patient outcomes [32]. In subgroup analysis, the higher pooled OR related to of European, followed by African and Middle East population. In the Far East, “HLA-B27” was a protective factor for developing BD.

Ultimately, according to the result this study, the chance for “HLA-B27” to develop BD differ among different countries or geographical areas with an increasing rate from East to West. In general, the result of numerous studies shows the difference in HLA-B27 distribution and its subtypes between population groups in worldwide [38–40] ranging from about 0% in Australian Aborigines, rare in the American black [8], 0.1–0.5% in Japanese, 2–9% in Chinese, 4% in North Africans and 8% in Caucasians [8, 38]. These differences are influenced by several factors such as ethnicity, geographic alterations, interaction with other genes, connection alleles with some forms of diseases, and environmental agent.

Moreover, migration from Asia to Europa or genetic drift and bottleneck effect, especially at the Middle East could express these changes [39]. In a study, Akassou was implied to relationship the HLA-B27 with high concentrations of testosterone in men [41]. In another study, Reveille JD in US observed a significantly higher odds for “HLA-B27” in younger than older adults [9]. Interaction between the HLA-B27 and foreign agents, such as human immunodeficiency virus (HIV), hepatitis C, Klebsiella, Shigella and Salmonella has been reported in a number of studies [9, 42]. In another study, Shimizu et al. reported the changes of microbes in the intestines of BD-patients with perdominity of Actinobacteria and Lactobacillus species following the stimulation of T helper 17 (Th17) cells [42].

Gene association studies displaying interaction of “HLA-B27” with ERAP1, ERAP2 and HLA-B60 genes [8, 43]. Also, the antigen of β2-GPI which lead to the presence of antiphospholipid antibodies has been considered in the development of autoimmune diseases [44].

It was clear that the accurate diagnosis of disease, improvement in the detection of pathogenic mutations in DNA and increased number of reports were going to have effect on the distribution of the “HLA-B27”.

### Strength and limitation

The advantage of the present study was the use of pooled OR for analysis and, comparison “HLA-B27” frequency between BD patients and controls for the first time.

This study was limited by incomplete information that prevented subgroup analysis for sex and age. Analysis of pooled OR for “HLA-B27”in subsets of BD or independent of HLA-B51/B5 is 10 recommended. As “HLA-B51”is the strongest associated with BD in variant ethnic populations, so it is suggested analyzing the frequency of “HLA-B27” independent from “HLA-B51”.

## Conclusion

Previous studies have been established the efficacy of HLA class I gens especially “HLA-B51” on BD. Based on the results of this study, the relationship between disease and “HLA-B27” could be explained. On the other hand, this study consider the impression of other HLA-B alleles (“HLA-B27”) on the risk of BD and the reinforcement of hypothesis Behcet disease–gene association. Fluctuations in risk ratios were explained due to the interconnection between environmental and genetic factors with “HLA-B27”. Future research should be conducted into the more evaluation of “HLA-B27” in BD, independent or concomitance of other agents, assessment the relationship this gene with clinical presentations and detection application these alleles in the management of disease.

## Abbreviations

BD: Behcet’s disease
AS: ankylosing spondylitis
SID: Scientific Information Database
IranDoc: Iranian Research Institute for Information Science and Technology
PRISMA: Preferred Reporting Items for Systematic Reviews
PICO: participants, interventions, comparison and outcome
OR: odds ratio
HLA: human leukocyte antigen
MHC: major histocompatibility complex
CMA: comparative meta-analysis

## References

[1] Remmers EF, Cosan F, Kirino Y, Ombrello MJ, Abaci N, Satorius C (2010). Genome-wide association study identifies variants in the MHC class I, IL10, and IL23R-IL12RB2 regions associated with Behcet’s disease. Nat Genet.

[2] Gul A (2001). Behcet’s disease: an update on the pathogenesis. Clin Exp Rheumatol.

[3] de Chambrun MP, Wechsler B, Geri G, Cacoub P, Saadoun D (2012). New insights into the pathogenesis of Behcet’s disease. Autoimmun Rev.

[4] Piga M, Mathieu AJR (2010). Genetic susceptibility to Behcet’s disease: role of genes belonging to the MHC region. Rheumatology.

[5] Radouane A, Oudghiri M, Chakib A, Naya A, Belhouari A, El Malki A (2011). HLA-B*27 allele associated to Behçet’s disease and to anterior uveitis in Moroccan patients. Ann Biol Clin.

[6] Gül A, Uyar F, Inanc M, Ocal L, Barrett J, Aral O (2002). A weak association of HLA-B* 2702 with Behçet’s disease. Genes Immun.

[7] Cohen R, Metzger S, Nahir M, Chajek-Shaul T (2002). Association of the MIC-A gene and HLA-B51 with Behcet’s disease in Arabs and non-Ashkenazi Jews in Israel. Ann Rheum Dis.

[8] Sheehan NJ (2004). The ramifications of HLA-B27. J R Soc Med.

[9] Reveille JD, Hirsch R, Dillon CF, Carroll MD, Weisman MH (2012). The prevalence of HLA-B27 in the US: data from the US National Health and Nutrition Examination Survey, 2009. Arthritis Rheum.

[10] Bettencourt A, Pereira C, Carvalho L, Carvalho C, Patto J, Bastos M (2008). New insights of HLA class I association to Behçet’s disease in Portuguese patients. Tissue Antigens.

[11] Shamseer L, Moher D, Clarke M, Ghersi D, Liberati A, Petticrew M (2015). Preferred reporting items for systematic review and meta-analysis protocols (PRISMA-P) 2015: elaboration and explanation. BMJ.

[12] Takano M, Miyajima T, Kiuchi M, Ohmori K, Amemiya H, Yokoyama T (1976). Behcet disease and the HLA system. Tissue Antigens.

[13] Chung YM, Tsai ST, Liao F, Liu JH (1987). A genetic study of Behcet’s disease in Taiwan Chinese. Tissue Antigens.

[14] Cibo H, Yi L, Ming CXX, Yuzhen D, Meng Q. Determination of HL-A antigens in Behcet’s disease. CNKI J. 1997;R597.9

[15] Brautbar C, Chajek T, Ben-Tuvia S, Lamm L, Cohen T (1978). A Genetic Study of Behqet Disease in Israel. Tissue Antigens.

[16] Yazici H, Chamberlain MA, Schreuder I, D’amaro J, Muftuoglu M (1980). HLA antigens in Behçet’s disease: a reappraisal by a comparative study of Turkish and British patients. Ann Rheum Dis.

[17] Müftuüǧlu A, Yazici H, Yurdakul S, Pazarli H, Özyazgan Y, Tüzün Y (1981). Behçs disease: lack of correlation of clinical manifestations with HLA antigens. Tissue Antigens.

[18] Pirim I, Atasoy M, Ikbal M, Erdem T, Aliagaoglu C (2004). HLA class I and class II genotyping in patients with Behcet’s disease: a regional study of eastern part of Turkey. Tissue Antigens.

[19] Mizuki N, Meguro A, Tohnai I, Gül A, Ohno S, Mizuki N (2007). Association of major histocompatibility complex class I chain-related gene A and HLA-B alleles with Behçet’s disease in Turkey. Jpn J Ophthalmol.

[20] Ombrello MJ, Kirino Y, de Bakker PI, Gül A, Kastner DL, Remmers EF (2014). Behcet disease-associated MHC class I residues implicate antigen binding and regulation of cell-mediated cytotoxicity. Proc Natl Acad Sci.

[21] Xavier JM, Shahram F, Davatchi F, Rosa A, Crespo J, Abdollahi BS (2012). Association study of IL10 and IL23R–IL12RB2 in Iranian patients with Behcet’s disease. Arthritis Rheum.

[22] Al-Okaily F, Al-Rashidi S, Al-Balawi M, Mustafa M, Arfin M, Al-Asmari A (2016). Genetic association of HLA-A* 26,-A* 31, and-B* 51 with Behcet’s disease in Saudi Patients. Clin Med Insights Arthritis Musculoskelet Disord.

[23] Mizuki N, Mahr A, Takeno M, Hirohata S (2017). Genome-wide association study for Behcet’s disease. Rheumatology.

[24] Chamberlain MA (1977). Behcet’s syndrome in 32 patients in Yorkshire. Ann Rheum Dis.

[25] Lehner T, Batchelor J, Challacombe S, Kennedy L (1979). An immunogenetic basis for the tissue involvement in Behçet’s syndrome. Immunology.

[26] Muñoz-Saá I, Cambra A, Pallarés L, Espinosa G, Juan A, Pujalte F (2006). Allelic diversity and affinity variants of MICA are imbalanced in Spanish patients with Behçet’s disease. Scand J Immunol.

[27] Montes-Cano MA, Conde-Jaldón M, García-Lozano JR, Ortiz-Fernández L, Ortego-Centeno N, Castillo-Palma MJ (2013). HLA and non-HLA genes in Behçet’s disease: a multicentric study in the Spanish population. Arthritis Res Ther.

[28] Choukri F, Chakib A, Himmich H, Marih L, Caillat-Zucman S (2003). HLA-B phenotype modifies the course of Behçet’s disease in Moroccan patients. Tissue Antigens.

[29] Sakly N, Boumiza R, Zrour-Hassen S, Hamzaoui A, Yahia SB, Amara H (2009). HLA-B27 and HLA-B51 Determination in Tunisian Healthy Subjects and Patients with Suspected Ankylosing Spondylitis and Behçet’s Disease. Ann NY Acad Sci.

[30] de Menthon M, Lavalley MP, Maldini C, Guillevin L, Mahr A (2009). HLA-B51/B5 and the risk of Behçet’s disease: a systematic review and meta-analysis of case–control genetic association studies. Arthritis Care Res.

[31] Meguro A, Inoko H, Ota M, Katsuyama Y, Oka A, Okada E (2010). Genetics of Behçet disease inside and outside the MHC. Ann Rheum Dis.

[32] Alpsoy E (2016). Behçet’s disease: a comprehensive review with a focus on epidemiology, etiology and clinical features, and management of mucocutaneous lesions. J Dermatol.

[33] Scholl HP, Fleckenstein M, Issa PC, Keilhauer C, Holz FG, Weber BH (2007). An update on the genetics of age-related macular degeneration. Molecular vision.

[34] McGonagle D, Aydin SZ, Gül A, Mahr A, Direskeneli H (2015). ‘MHC-I-opathy’—unified concept for spondyloarthritis and Behçet disease. Nat Rev Rheumatol.

[35] Takeuchi M, Kastner DL, Remmers EF (2015). The immunogenetics of Behçet’s disease: a comprehensive review. J Autoimmun.

[36] Gül A, Hajeer AH, Worthington J, Barrett JH, Ollier WE, Silman AJ (2001). Evidence for linkage of the HLA-B locus in Behçet’s disease, obtained using the transmission disequilibrium test. Arthritis Rheum Off J Am Coll Rheumatol.

[37] Savey L, Resche-Rigon M, Wechsler B, Comarmond C, Piette JC, Cacoub P (2014). Ethnicity and association with disease manifestations and mortality in Behçet’s disease. Orphanet J Rare Dis.

[38] Nessa A, Tabassum S, Sultana S (2014). HLA-B27 antigen frequency among suspected Spondyloarthropathy patients attaining a tertiary level hospital of Bangladesh. Bangladesh Med Res Counc Bull.

[39] García-Fernández S, Gonzalez S, Miña Blanco A, Martinez-Borra J, Blanco-Gelaz M, López-Vazquez A (2001). New insights regarding HLA-B27 diversity in the Asian population. Tissue Antigens.

[40] Ball EJ, Khan MA (2001). HLA-B27 polymorphism. Joint Bone Spine.

[41] Akassou A, Bakri Y (2018). Does HLA-B27 status influence ankylosing spondylitis phenotype?. Clin Med Insights Arthritis Musculoskelet Disord.

[42] Shimizu J, Kubota T, Takada E, Takai K, Fujiwara N, Arimitsu N (2016). Bifidobacteria abundance-featured gut microbiota compositional change in patients with Behcet’s Disease. PLoS ONE.

[43] Vitulano C, Tedeschi V, Paladini F, Sorrentino R, Fiorillo MT (2017). The interplay between HLA-B27 and ERAP1/ERAP2 aminopeptidases: from anti-viral protection to spondyloarthritis. Clin Exp Immunol.

[44] Chao Y, Chen D-Y, Lan J-L, Tang K-T, Lin C-C (2018). Tolerogenic β2-glycoprotein I DNA vaccine and FK506 as an adjuvant attenuates experimental obstetric antiphospholipid syndrome. PLoS ONE.

